# Risk factors for therapeutic failure to meglumine antimoniate and miltefosine in adults and children with cutaneous leishmaniasis in Colombia: A cohort study

**DOI:** 10.1371/journal.pntd.0005515

**Published:** 2017-04-05

**Authors:** Maria del Mar Castro, Alexandra Cossio, Carlos Velasco, Lyda Osorio

**Affiliations:** 1 Centro Internacional de Entrenamiento e Investigaciones Médicas (CIDEIM), Cali, Colombia; 2 Escuela de Salud Pública, Universidad del Valle, Cali, Colombia; 3 Departamento de Pediatría, Universidad del Valle, Cali, Colombia; Ohio State University, UNITED STATES

## Abstract

**Introduction:**

Reports of therapeutic failure to meglumine antimoniate (MA) and miltefosine in cutaneous leishmaniasis (CL) varies between species, populations and geographic regions. This study aimed to determine the clinical, drug-related factors, and *Leishmania* species associated with treatment failure in children and adults with cutaneous leishmaniasis.

**Methods:**

A cohort study was performed with children (2–12 years old) and adults (18–65 years old) with CL, who have participated in clinical studies at CIDEIM Cali, Tumaco and Chaparral. Incidence of therapeutic failure was estimated by treatment and age groups. Descriptive, bivariate, and multiple logistic regression analyses were performed for the complete cohort and pediatric patients.

**Results:**

Two hundred and thirty patients were included (miltefosine: 112; MA: 118), of which 60.4% were children and 83.9% were infected with *L*.*V*. *panamensis*. Overall incidence of therapeutic failure was 15.65% (95%CI: 10.92–20.38), and was lower for miltefosine than for MA (8.92%, 95%CI: 3.59–14.26 versus 22.03%, 95%CI:14.48–29.58, p = 0.006). Treatment failure was associated with age ≤8 years (OR: 3.29; 95%CI: 1.37–7.89), disease duration ≤1 month (OR: 3.29; 95%CI: 1.37–7.89), regional lymphadenopathy (OR: 2.72; 95%CI: 1.10–6.70), treatment with MA (OR: 3.98; 95%CI: 1.66–9.50), and adherence <90% (OR: 3.59; 95%CI: 1.06–12.11). In children, higher Z-score of height/age was a protective factor (OR: 0.58; 95%CI: 0.36–0.93), while treatment with MA was a risk factor (OR: 40.82; 95%CI: 2.45–677.85), demonstrating significant interaction with age (p = 0.03).

**Conclusions:**

Clinical and drug-related factors determine therapeutic failure in CL. High risk of failure in children treated with MA indicates the need to reconsider this drug as first line treatment in this population.

**Trial registration:**

Clinical trial registration: NCT00487253

Clinical trial registration: NCT01462500

Clinical trial registration: NCT01464242

## Author summary

Cutaneous leishmaniasis (CL) is a parasitic disease that causes chronic, often ulcerated, skin lesions. Treatment require administration of systemic and poorly tolerated drugs, of which, the most commonly used is meglumine antimoniate (MA) injections during 20 days. Although children and adults might have different responses to these drugs, Colombian treatment guidelines recommends meglumine antimoniate for all age groups. In this study, we explored the factors that influence the therapeutic response in children and adults with CL treated with MA and miltefosine. We included 230 children and adults in the analysis, and we found that young age (≤8 years old), presence of regional lymphadenopathy, disease duration ≤1 month, poor adherence to treatment (<90%) were associated with increased odds of treatment failure. Additionally, being treated with MA was a risk factor for therapeutic failure, especially for children. A better nutritional status (higher Z-score of height/age) was a protector factor in pediatric patients. These results highlight the urgent need to reconsider MA as first line treatment in children in Colombia and to evaluate better treatment options for this population.

## Introduction

Cutaneous leishmaniasis is a public health problem with the number of cases annually ranging from 0.7–1.2 million worldwide [1]. Seventy-five percent of the global burden of disease is concentrated in ten countries, including Colombia, where cutaneous leishmaniasis is an important cause of morbidity [1,2]. In the Americas, cutaneous leishmaniasis is caused principally by species of the *Viannia* subgenus. Clinical manifestations range from mild, localized, self-healing lesions to numerous disseminated cutaneous lesions, severe chronic cutaneous and mucosal lesions. Spontaneous cure of cutaneous lesions, frequently leaving scars, occurs in less than 30% of cases, of which 20% result in relapse after initial healing [2].

Treatment involves administration of often toxic and poorly tolerated drugs [3]. For over 5 decades, first line treatment for all age groups has been parenteral antimonial drugs whose efficacy varies across regions, age groups, and *Leishmania* species. Treatment success with these drugs can be as low as 25% in children younger than 5 years old [4, 5]. Another available and recommended option is oral miltefosine, which has comparable efficacy to meglumine antimoniate in children [6]; its cure rates also vary by species and geographic location [6–9], and is as low as 60% in military populations [7]. Second line options include amphotericin B and pentamidine, which are more toxic and require parenteral administration [5, 10].

The identification of determinants of treatment response in American cutaneous leishmaniasis provides the basis for determining high risk patients, to orient the selection of treatment regimens, and to design interventions for those factors that are modifiable. Together these measures contribute to the preservation of the useful life of current medications. Known determinants of treatment outcome in cutaneous leishmaniasis include adherence to the treatment, *Leishmania* species, number and location of lesions, duration of the disease, and age [11–13]. However, the studies that have identified these associations have focused on adult patients and treatment with antimonials. Consequently, little is known about factors associated with treatment response in pediatric populations, or regarding treatment with miltefosine. This study sought to determine the clinical and drug-related factors, and *Leishmania* species associated with treatment failure in children and adults with cutaneous leishmaniasis.

## Methods

### Ethics statement

This study was approved and monitored by Universidad del Valle (approval number: 012–014) and CIDEIM’s institutional review boards for ethical conduct of research involving human subjects, and followed national and international clinical research ethics guidelines. Waiver of informed consent for the use of data was requested and accepted.

### Study design

We designed a cohort study using secondary data from four clinical studies conducted by investigators and collaborators of the Centro Internacional de Entrenamiento e Investigaciones Medicas (CIDEIM) between 2007 and 2013 in three municipalities of Colombia. The largest study was a non-inferiority trial (Clinical trial registration: NCT00487253) comparing miltefosine and pentavalent antimony in children; the second was a pharmacokinetic trial of miltefosine in children and adults (Clinical trial registration: NCT01462500), the third was an immunologic study of patients treated with pentavalent antimony, and the smallest was an add-on trial evaluation of pentoxifylline or placebo to the antimonial treatment (Clinical trial registration: NCT01464242).

### Study setting and participants

The participants were recruited within the original studies on an outpatient basis in three municipalities in the central and southwestern regions of Colombia: Tumaco (2°48´24” N, 78°45´53” W), which is located in the southern Pacific coast of Colombia and is an endemic region for *L*. *panamensis* and *L*. *braziliensis*; Cali (3°26′13″ N; 76°31′20″ W), which is a referral center for cases of cutaneous leishmaniasis from the southwestern region of the country, and Chaparral (3°43′23″ N; 75°28′59″ W), which is an endemic area located in the central region of Colombia and the site of a recent epidemic of cutaneous leishmaniasis caused by *L*. *guyanensis* [6].

Eligible participants in the clinical studies were children aged 2–12 years or adults aged 18–60 years with parasitologically confirmed cutaneous leishmaniasis (positive direct smear, culture of lesion aspirates or biopsy, as described in the original studies). Parasite identification was performed using subgenus and species discriminating monoclonal antibodies [14]. For the present study, those who received treatment with pentavalent antimony or miltefosine and completed the study follow-up scheme (minimum 13 weeks or 90 days if pentavalent antimony, and minimum 26 weeks or 180 days if miltefosine) were included. Patients’ records that did not include assessment of therapeutic response or have missing data regarding weight or treatment information (doses prescribed and received) were excluded. All patients who met eligibility criteria were included in the analysis.

### Outcome and exposure measures

The main outcome of this study is therapeutic failure, determined at or before 26 weeks following initiation of treatment, according to the criteria described by Rubiano and colleagues [6]. Cure was defined as complete re-epithelization and the absence of inflammatory signs for all cutaneous leishmaniasis lesions at day 90 (week 13), and maintained until the end of the follow-up. Therapeutic failure was defined as incomplete re-epithelization and/or the presence of induration, raised borders, or redness in any lesion after day 90, relapse (reactivation of lesions after initial cure) or the appearance of new lesions. Exposure variables were classified as related to the host, drug or parasite, including age, sex, number, duration and location of lesions, concomitant adenopathy, *Leishmania* species, dose and type of medication. Height and weight data were used to calculate Z-scores in pediatric patients, using the Anthro plus software [15]. All variables were measured at baseline, except for the adherence to the antileishmanial drug which was defined at the end of treatment as the proportion of received doses over the total doses prescribed. We considered as compliant those patients with adherence ≥90%.

Outcome and exposure variables were obtained from databases of the original studies, which followed similar operation procedures for measurement of these variables. All studies controlled adherence using diaries and product count. None of the outcome measurements were masked.

### Statistical analysis

Overall risk of therapeutic failure was estimated for the complete cohort. Quantitative exposure variables were compared using Student´s T test or Mann–Whitney test according to normal or skewed distribution, respectively. For qualitative variables, the Chi-squared test or Fisher´s exact test were used when appropriate. Relative risks and their corresponding 95% confidence intervals were calculated. Multiple logistic regression modeling was used to estimate the Odds Ratio of therapeutic failure using the backward selection technique; a p-value <0.05 was considered statistically significant. Interactions between treatment, age, and *Leishmania* species were assessed in the final model.

Subgroup analysis was performed in children (≤12 years old) and in a group of patients who completed follow-up to 26 weeks, as part of a sensitivity analysis, in order to evaluate the change in the OR when participants with follow-up to 13 weeks were removed from the model. This was used to evaluate the presence of outcome misclassification in patients who were treated with meglumine antimoniate and did not have follow-up at 26 weeks (study number 3). Age categories (≤8 years old vs >8 years old) were used in the analysis of the complete cohort, considering reported differences in efficacy of meglumine antimoniate in children [6]; however, age was analyzed as a continuous variable for subgroup analysis in pediatric patients. Goodness of fit test, Area Under the Curve (AUC), and Akaike information criteria were used for model selection. All the analyses were performed using STATA 10.

## Results

**Fig 1:**
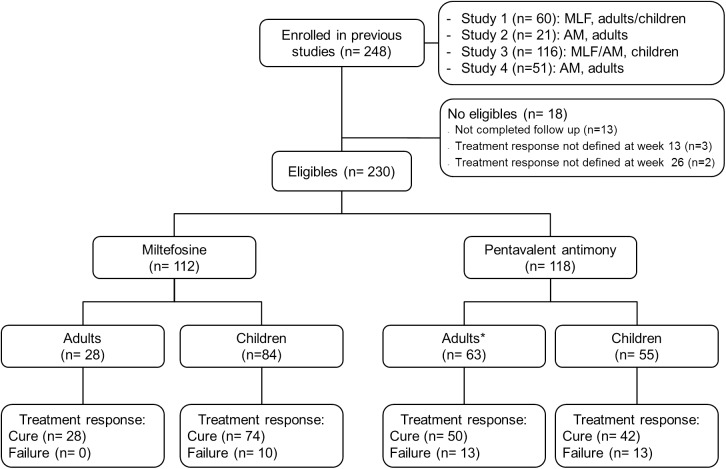
Inclusion and follow up of study participants. * All 63 adult patients have therapeutic response data at week 13, but 28 had therapeutic response data at 26 weeks. Imputation of therapeutic response based on 13 week’s response was performed for these patients.

A total of 248 patients were enrolled in the four clinical studies, of which 230 were eligible for the cohort study (Fig 1). The age distribution ranged from 2 to 60 years old, with a mean of 10 years old. Ninety-five were female (41.3%) and 78.4% came from the Pacific region of Colombia. Species identification of *Leishmania* parasites was available in 62.1% of participants, of which 83.9% were *L*. *V*. *panamensis*.

Regarding the clinical presentation, 84.6% of lesions were ulcers, with median diameter of 22mm (IQR: 14 –32), located mainly on the arms and legs (87.1%). The median duration of the oldest lesion was 2 months (IQR: 1–3), and the median number of lesions was 1 (IQR: 1–3). Fifty-one percent of patients received treatment with miltefosine. The proportion of non-adherence was 9.5% (<90% adherence, 22 patients) for all treatment regimens taken together. The proportion of non-adherence was higher in the group treated with antimonial versus miltefosine (14.41% vs 4.46%, p = 0.009).

Descriptive statistics and relative risk of failure were estimated (Table 1). Overall incidence of therapeutic failure was 15.6% (95%CI: 10.92–20.38), with a higher incidence of failure in patients treated with pentavalent antimony compared to miltefosine (22.03%, 95% CI: 14.48–29.58 vs. 8.93, 95%CI: 3.59–14.26), p = 0.006. In the univariate analysis, therapeutic failure was associated with duration of the oldest lesion being less than 1 month (RR: 1.94, 95%CI: 1.06–3.54), regional lymphadenopathy (RR: 1.94, 95% CI: 1.02–3.68), and treatment with pentavalent antimony (RR: 2.46; 95%CI: 1.24–4.88). Height/age Z-score was significantly lower in children with therapeutic failure than children who were cured (p = 0.04). The analyses did not reveal an association between treatment outcome and *Leishmania* species, previous episodes of leishmaniasis, other clinical or socio-demographic variables. There was no difference in the incidence of failure by year of recruitment (p = 0.46).

**Table 1:** Incidence and relative risk of treatment failure according to host, parasite and drug-related characteristics.

Characteristics	Cure	Failure	RR	95% CI	p
	n = 194	n = 36			
**Related to the host**					
Age, years. Me (IQR)	10 (7–29)	8 (4.5–28.5)			0.12^§^
Age. No (%)					
≤8 years	75 (78.95)	20 (21.05)	1.78	0.97–3.25	0.05
>8 years	119 (88.15)	16 (11.85)	1		
Sex. No (%)					
Male	115 (85.19)	20 (14.81)	0.87	0.48–1.61	0.67
Female	79 (83.16)	16 (16.84)	1		
History of leishmaniasis. No (%)					
Yes	14 (87.50)	2 (12.50)	0.78	0.20–2.96	0.71
No	168 (84.00)	32 (16.00)	1		
Previous antimonial treatment. No. (%)					
Yes	14 (87.50)	2 (12.50)	0.78	0.20–2.96	0.71
No	168 (84.00)	32 (16.00)	1		
Study site. No. (%)					
Cali	46 (88.46)	6 (11.54)	0.66	0.28–1.54	0.33
Tumaco	110 (82.71)	23 (17.29)	1		
Chaparral	38 (84.44)	7 (15.56)	0.89	0.41–1.95	0.78
Region of origin of the case. No. (%)					
Pacific	146 (85.38)	25 (14.62)	1		
Andeans	35 (83.33)	7 (16.67)	1.14	0.52–2.45	0.73
Other	5 (100)	0	-		
Weight. Kg. Me (IQR)	30.4(20.8–65.2)	21.35 (17–62.85)			0.07^§^
Clinical presentation					
Duration oldest lesion (months). Me (IQR)	2 (1–3)	1 (1–2)			0.02^§^
Duration oldest lesion (months). No (%)					
≤1	70 (77.78)	20 (22.22)	1.94	1.06–3.54	0.02
>1	124 (88.57)	16 (11.43)	1		
Nutritional status	(n = 115)	(n = 23)			
Z score BMI/age. Media (SD)	-0.01 (0.97)	-0.12 (1)			0.62^†^
Z score height/age. Media (SD)	-0.78 (0.96)	-1.27 (1.47)			0.04^†^
Characteristics of cutaneous lesions					
Number of lesions. Me (IQR)	1 (1–3)	2 (1–3)			0.18^§^
Number of lesions. No (%)					
≤2	144 (86.23)	23 (13.77)	1		0.20
>2	50 (79.37)	13 (20.63)	1.49	0.80–2.77	
Total lesion area, mm^2^. Me (IQR)	710.39 (323.58–1338.25)	723.12 (416.82–1239.75)			0.82^§^
Diameter of the largest lesion, mm. Me (IQR)	28 (20.12–39.5)	26 (19.5–41)			0.65^§^
Type of lesion. No (%)					
Ulcer	173 (83.98)	33 (16.02)	1.28	0.42–3.86	0.65
Non-ulcerated lesion	21 (87.50)	3 (12.50)	1		
Regional lymphadenopathy. No. (%)					
Yes	28 (73.68)	10 (26.32)	1.94	1.02–3.68	0.04
No	166 (86.46)	26 (13.54)	1		
Lymphatic tract. No. (%)					
Yes	11 (78.57)	3 (21.43)	1.40	0.49–4.01	0.53
No	183 (84.72)	33 (15.28)	1		
Lesions on head—neck No (%)					
Yes	56 (83.58)	11 (16.42)	1.07	0.55–2.04	0.83
No	138 (84.66)	25 (15.34)	1		
Lesions on trunk No (%)					
Yes	30 (81.08)	7 (18.92)	1.25	0.59–2.65	0.55
No	164 (84.97)	29 (15.03)	1		
Lesions on arms No (%)					
Yes	101 (80.80)	24 (19.20)	1.68	0.88–3.19	0.10
No	93 (88.57)	12 (11.43)	1		
Lesions on legs. No (%)					
Yes	58 (87.88)	8 (12.12)	0.70	0.34–1.47	0.34
No	136 (82.93)	28 (17.07)	1		
Concomitant distal lesions. No (%)					
Yes	37 (75.51)	12 (24.49)	1.84	0.99–3.42	0.05
No	157 (86.74)	24 (13.26)	1		
**Related to the parasite (n = 143)**					
*Leishmania* species. No (%)	n = 119	n = 24			
*L*. *V*. *panamensis*	99 (82.50)	21 (17.50)	1		
*L*. *V*. *braziliensis*	5 (83.33)	1 (16.67)	0.95	0.15–5.94	0.95
*L*. *V*. *guyanensis*	9 (100)	0 (0)	-		
Other**	6 (75.00)	2 (25.00)	1.42	0.40–5.04	0.59
**Related to the drug (n = 230)**					
Prescribed drug. No (%)	n = 194	n = 36			
Meglumine antimoniate	92 (77.97)	26 (22.03)	2.46	1.24–4.88	0.006
Miltefosine	102 (91.07)	10 (8.93)	1		
Completed ≥90% treatment. No (%)					
Yes	178 (85.58)	30 (14.42)	1		0.11
No	16 (72.73)	6 (27.27)	1.89	0.88–4.03	
**Miltefosine treatment**	n = 102	n = 10			
Adherence percentage. Me (Range)	100 (10.7–100)	100 (85.7–100)			0.35^§^
Prescribed dose (mg/Kg/day). Me (IQR)	2.27 (2.06–2.35)	2.31 (2.3–2.4)			0.15^§^
**Meglumine antimoniate**	n = 92	n = 26			
Adherence percentage. Me (Range)	100 (50–100)	100 (36.6–100)			0.51^§^
Prescribed dose (mg/Kg/day). Me (IQR)	19.98 (19.79–20.27)	19.95 (19.63–20.35)			0.55^§^

^§^U-Mann-Whitney test.

^†^t-test.

** *L*. *Viannia* isolates, not classified up to species level, one patient with *L*. *Mexicana*.

Factors independently associated with treatment failure included: age ≤ 8 years old (aOR: 3.29; 95%CI: 1.37–7.89), duration of the oldest cutaneous lesion ≤ 1 month (aOR: 2.85; 95%CI: 1.29–6.28), regional lymphadenopathy (aOR: 2.72; 95%CI:1.10–6.70), treatment with meglumine antimoniate (aOR: 3.98; 95%CI: 1.66–9.50), and less than 90% adherence to the treatment (aOR: 3.59; 95%CI: 1.06–12.11) (Table 2). No association of therapeutic response was evident with the *Leishmania* species isolated from the patient, and we did not find significant interactions between treatment, age, or species. However, given that 62.1% of patients had data regarding the identity of the infecting species, a model including this variable diminishes the sample size to 58% of the overall cohort, hence, statistical power to make inferences regarding parasite species.

**Table 2:** Factors associated with therapeutic failure in the cohort (n = 230).

Factor	Crude OR			Adjusted OR		
	OR	95% CI	p	OR	95% CI	p
Treatment with meglumine antimoniate	2.88	1.31–6.30	0.008	3.98	1.66–9.50	0.002
Adherence to the treatment <90%	2.22	0.80–6.13	0.12	3.59	1.06–12.11	0.039
Age ≤ 8 years old	1.98	0.96–4.06	0.06	3.29	1.37–7.89	0.007
Duration of oldest lesion ≤1 month	2.21	1.07–4.54	0.03	2.85	1.29–6.28	0.009
Regional lymphadenopathy	2.28	0.99–5.23	0.05	2.72	1.10–6.70	0.029

In the pediatric population (Table 3), we found that nutritional status, represented by the Z-score of height/age, was a predictor of treatment response because the odds of failure decreased 48% for every unit of increase in the Z-score (aOR: 0.55; 95%CI: 0.31–0.86). Presence of regional lymphadenopathy was also identified as a risk factor in this group. Treatment with pentavalent antimony in children showed a statistically significant association with failure, more so than in the rest of the overall study population (aOR: 40.82; 95% CI: 2.45–677.85).

**Table 3:** Factors associated with therapeutic failure in the pediatric population (n = 138).

Factor	Crude OR			Adjusted OR		
	OR	95% CI	p	OR	95% CI	p
Treatment with meglumine antimoniate	2.29	0.92–5.67	0.07	40.82	2.45–677.85	0.01
Regional lymphadenopathy	3.33	1.21–9.12	0.01	5.98	1.67–21.37	0.006
Duration of oldest lesion ≤1 month	1.98	0.79–4.94	0.14	3.20	1.08–9.45	0.03
Z-score height/age	0.66	0.44–1.00	0.05	0.52	0.31–0.86	0.01
Age (years) x meglumine antimoniate				0.63	0.41–0.96	0.03
Age (years)	0.83	0.71–0.99	0.04	0.99	0.77–1.27	0.95

A significant interaction between age and treatment with meglumine antimoniate was found, where the odds of treatment failure decreased for each additional year in patients treated with this drug (aOR: 0.63; 95% CI: 0.41–0.96, p = 0.03), as opposed to those patients treated with miltefosine (effect of age: aOR: 0.99; 95% CI: 0.77–1.27). We found neither a statistically significant association nor interaction between *Leishmania* species and the study outcome.

The models shown in Tables 2 and 3 fit the data. The first model (Table 2) with p = 0.29 in the Goodness of fit test and the area under the ROC curve (AUC) = 0.76 indicates a good data discrimination; the second model (Table 3) had estimated values of p = 0.49 and AUC = 0.81. Sensitivity analysis showed that after excluding the patients without follow-up at 26 weeks (n = 43), the explanatory variables in the model remained statistically significant, although the aOR of age ≤ 8 years and adherence increased to 8.37 (95% CI: 2.16–32.41) and 19.36 (95% CI: 3.14–119.15), respectively (S1 Table).

## Discussion

This study assessed risk factors for therapeutic failure in children and adults with parasitologically confirmed cutaneous leishmaniasis in Colombia. The overall incidence of therapeutic failure was 15.65% (95% CI: 10.92–20.38), which was higher with pentavalent antimony than miltefosine [16]. Estimates of treatment failure with meglumine antimoniate (22.03%, 95%CI: 14.48–29.58) are similar to previous reports from Colombian studies [6, 17], although they are lower than reported in other regions of Latin America [11, 12]. In this cohort, the proportion of treatment failure with miltefosine (8.92%, 95%CI: 3.59–14.26) was lower than reported in other studies conducted in children and adults [6, 7], although it was similar to the proportion reported by Soto *et al* in patients with *L*. *panamensis* infection in other regions of Colombia [8]. The lower proportion of treatment failure can partially be explained by the characteristics of this cohort of patients being enrolled in clinical studies [18], under supervised or directly-observed treatment, which are interventions that have shown a positive effect regarding the therapeutic response to antimicrobials [19].

Due to the inclusion of patients with either pentavalent antimony or miltefosine medications, we were able to identify antimonial treatment as an independent risk factor for therapeutic failure (OR: 3.98; 95%CI: 1.66–9.50). This drug has been the first line treatment for over 70 years, with its efficacy ranging from 70–85% [6, 20]. Although the efficacy of meglumine antimoniate in this study falls in this range, the efficacy of miltefosine is higher. Possible explanation of the high cure rate of miltefosine is related to the predominance of one *Leishmania* species in the study and its known parasite susceptibility profile. Variations in parasite susceptibility to antileishmanial drugs have previously been reported by species and geographic locations [5, 6, 20]. In this study, the largest proportion of participants were infected in the pacific coast of Colombia, where *L*.*V*. *panamensis* is predominant and has shown a better *in vitro* susceptibility to miltefosine compared with other species isolated from the eastern parts of the Andean and Orinoco regions of the country [21], explaining, at least partially, the good clinical response to miltefosine.

Age under or equal to 8 years old was identified as a predictor of failure in the complete cohort, independent of the other factors (OR: 3.59; 95%CI: 1.06–12.11). Among children, younger age was also associated with increased odds of therapeutic failure. This finding is consistent with previous studies [4, 11, 12]. Differences in the pharmacokinetics of antileishmanial drugs in children are a possible explanation. Clearance of pentavalent antimony in children is faster than in adults, and therefore the maximum plasma concentration (Cmax) and the Area Under the Curve (AUC) of the drug are lower compared to adults [22]. Regarding miltefosine, analysis using Monte Carlo simulations of PK data from patients with visceral leishmaniasis have described lower plasma concentrations in children under the current linear dosing regimen (mg/kg) [23]. Additionally, reports of a clinical trial aimed at evaluating the pharmacokinetics of miltefosine in children shows lower Cmax and AUC in children at the same dose regimen (clinicaltrials.org number: NCT01462500) [24]. This is important because miltefosine seems to be a time-dependent antimicrobial drug, and the risk of failure in patients with visceral leishmaniasis is increased with the number of days below 10xEC_50_ miltefosine plasma concentrations [25].

Age effect also varies according to the prescribed drug in the study population, as shown with a statistically significant interaction between age and meglumine antimoniate (p = 0.03). This suggests that antimonial treatment in children is related to treatment failure, and is concordant with differences in treatment efficacy by age groups, as previously described [4]. Allometric dosing of miltefosine have been proposed as an alternative to improve drug exposure in children, and its safety is being evaluated currently for VL patients (NCT02431143 and NCT02193022); however, little is known about feasibility or safety of alternative dosing regimens for antimonials. Despite these facts, meglumine antimoniate is still the first line treatment in all age groups in Colombia, which highlights the urgent need to reconsider the management of pediatric cutaneous leishmaniasis with improved dosing or alternative treatments [26].

Partial immunity attributed to antigen exposition in endemic areas and differences in immune response in children can be related to the effect of age on treatment response, as described in other parasitic infections [27]. This hypothesis is supported by the increment of prevalence of *Leishmania* infection by age groups in this endemic area [28], which might imply a lower exposure to *Leishmania* in younger children. Another determinant of therapeutic outcome was disease duration. In cutaneous leishmaniasis, early treatment has been described as a risk factor for treatment failure [11, 12], and management with antimonials before 20 days of disease appearance did not prevent the ulceration of lesions, therefore being associated with worse prognosis [29, 30]. This is explained partially by the role of immune response in treatment outcomes [30], when patients are treated before reaching protective acquired immunity. Surrogates of immune response such as the diameter of Montenegro skin test (MST), gamma interferon (INF-γ), and TNF-α (in supernatant of cell cultures) were reduced in patients with early treatment in a previous study [29]. Additionally, induration of MST was described as a determinant of therapeutic outcome in CL in a recent study, where short duration of the disease was another risk factor for failure (OR: 6.33; 95%CI: 2.52–15.90), supporting the impact of variables related to the host immunity in response to medications [31].

Poor treatment adherence and regional lymphadenopathy were independent factors for failure. Among these, irregular treatment was reported by Rodriges et al. as a determinant of poor outcome in CL, (RR: 1.85; 95%CI:1.33–2.56) [13], and is probably related to the lower drug exposure in this group of patients. Regional lymphadenopathy is an early sign of *Leishmania* infection, especially *L*. *V*. *braziliensis*, which could be present before the ulceration of cutaneous lesions [30, 32]. In this study, presence of lymphadenopathy was independent of disease duration (presence of lymphadenopathy in patients with disease duration ≤1 month: 34.2% vs >1 month: 65.8%, p = 0.49). Therefore, we hypothesized that it can be an indicator of disease severity or lymphatic dissemination of parasites, as described elsewhere [33, 34]. It may also be an indicator of the relationship between the reticuloendothelial system as a site of parasite persistence [35], although the nature of that relationship remains still unclear [35, 36]. This is a new finding that warrants further investigation.

Higher height/age z-score values in children were associated with a decrease in the odds of treatment failure (OR: 0.52; 95%CI: 0.31–0.86), suggesting that a better nutritional status is a protective factor. Low height/age z-scores can be used to identify children at risk of stunting [37, 38], and is a robust measure for population nutritional studies [39, 40]. Few studies have confirmed the relationship between malnutrition and risk of *Leishmania* infection [41, 42] and a small study in adults did not find a relationship between weight and time-to-cure in tegumentary leishmaniasis [43]. However, using this indicator, we provide evidence of the influence of nutrition on treatment response in children with CL, as has been described in other infectious diseases [44, 45], murine models of leishmaniasis [46], and in a small descriptive study conducted in children treated with miltefosine [47]. Possible explanations include the negative effect of malnutrition in delayed immune response, due to deficiency in vitamins A, C, E and minerals such as Zinc, among other factors [44, 45].

Influence of parasite species on clinical outcome is shown in several studies [5, 11, 12], but we were unable to identify this association. Low proportion of isolates (62%) and the predominance of *L*. *V*. *panamensis* species in over 83% of participants can explain this lack of association, which is a limitation of this study. Another important limitation of this study is the imputation of data from treatment outcomes in participants treated with meglumine antimoniate with follow-up at 13 weeks (90 days), which represents the end of follow-up in one of the studies included in the analysis. In order to measure its impact, we performed a sensitivity analysis excluding these patients, and the risk factors remained significant with similar force of association (S1 Table). Moreover, attendance to follow-up visits can be as low as 5% at six months, which is probably the most common scenario in monitoring treatment response in CL due to barriers to accessing health facilities in endemic areas.

Despite these limitations, the standardized measurement of treatment outcome, adherence, and baseline characteristics allows for comparisons between subjects, and overcomes some restrictions involved with assessing risk factors based on routine data. Moreover, the proportion of pediatric patients is large (60%), which enables stronger inferences in this group compared with other studies of treatment failure in CL. Although data were collected in clinical studies, we included different sites from two endemic areas of the country, which allowed us to generalize our findings to CL patients from central and southwestern Colombia with predominance of *L*. *V*. *panamensis*.

In conclusion, we provide evidence regarding the risk factors for treatment failure in adult and pediatric populations with cutaneous leishmaniasis. Modifiable characteristics, including timing of treatment, nutritional status, and use of antimonials, were identified as potential interventions to improve therapeutic success. The study highlights the urgent need to reconsider pentavalent antimony as first line treatment in children in Colombia where *L*. *V*. *panamensis* predominates.

## Supporting information

S1 Checklist STROBE Checklist. (PDF)

S1 Table Sensitivity analysis of treatment response at 13 and 26 weeks. (DOCX)
