# Supplementary material for: Risk factors for therapeutic failure to meglumine antimoniate and miltefosine in adults and children with cutaneous leishmaniasis in Colombia: A cohort study
Source: PLoS Negl Trop Dis. 2017 Apr 5;11(4):e0005515. doi: 10.1371/journal.pntd.0005515 (PMC5393627; doi:10.1371/journal.pntd.0005515)
Supplement: S1 Checklist — (PDF) [file pntd.0005515.s001.pdf]

S2. STROBE checklist of items that should be included in reports of observational studies

	Item No.	Recommendation	Page No.	Relevant text from manuscript
**Title and abstract**	1	(*a*) Indicate the study's design with a commonly used term in the title or the abstract	1, 2	The title states "a cohort study", and this is also described in the abstract
		(*b*) Provide in the abstract an informative and balanced summary of what was done and what was found	2	This is described in the abstract
**Introduction**				
Background/rationale	2	Explain the scientific background and rationale for the investigation being reported	5, 6	This is described in the introduction
Objectives	3	State specific objectives, including any prespecified hypotheses	6	"This study sought to determine the clinical and drug-related factors, and *Leishmania* species associated with treatment failure in children and adults with cutaneous leishmaniasis"
**Methods**				
Study design	4	Present key elements of study design early in the paper	6	Section "study design"
Setting	5	Describe the setting, locations, and relevant dates, including periods of recruitment, exposure, follow-up, and data collection	7	Section "Study setting and participants"
Participants	6	(*a*) *Cohort study*—Give the eligibility criteria, and the sources and methods of selection of participants. Describe methods of follow-up<br>*Case-control study*—Give the eligibility criteria, and the sources and methods of case ascertainment and control selection. Give the rationale for the choice of cases and controls<br>*Cross-sectional study*—Give the eligibility criteria, and the sources and methods of selection of participants	7	Section "Study setting and participants"
		(*b*) *Cohort study*—For matched studies, give matching criteria and number of exposed and unexposed<br>*Case-control study*—For matched studies, give matching criteria and the number of controls per case		Not applicable

Continued on next page

Variables	7	Clearly define all outcomes, exposures, predictors, potential confounders, and effect modifiers. Give diagnostic criteria, if applicable	7, 8	Section: “Outcome and exposure measures”
Data sources/ measurement	8*	For each variable of interest, give sources of data and details of methods of assessment (measurement). Describe comparability of assessment methods if there is more than one group	6 - 8	Described in sections: “Study setting and participants” and “Outcome and exposure measures”
Bias	9	Describe any efforts to address potential sources of bias	8, 9	Section: “Statistical analysis”
Study size	10	Explain how the study size was arrived at		As the number of patients was determined by the size of the original studies, all patients who met eligibility criteria were included in the analysis.
Quantitative variables	11	Explain how quantitative variables were handled in the analyses. If applicable, describe which groupings were chosen and why	8, 9	Section: “Statistical analysis”
Statistical methods	12	(*a*) Describe all statistical methods, including those used to control for confounding	8, 9	Section: “Statistical analysis”
		(*b*) Describe any methods used to examine subgroups and interactions	9	Section: “Statistical analysis”
		(*c*) Explain how missing data were addressed	7, 9	“Patients’ records that did not include assessment of therapeutic response or have missing data regarding weight or treatment information (doses prescribed and received) were excluded.” Additional information is described in the section of “Statistical analysis”.
		(*d*) *Cohort study*—If applicable, explain how loss to follow-up was addressed<br>*Case-control study*—If applicable, explain how matching of cases and controls was addressed<br>*Cross-sectional study*—If applicable, describe analytical methods taking account of sampling strategy		Not applicable: only participants with complete follow-up in the original studies were included in this analysis. See section “Study setting and participants”
		(*e*) Describe any sensitivity analyses	9	Section: “Statistical analysis”
**Results**				
Participants	13*	(a) Report numbers of individuals at each stage of study—eg numbers potentially eligible, examined for eligibility, confirmed eligible, included in the study, completing follow-up, and analysed	9, 10	This is described in the results section and figure 1
		(b) Give reasons for non-participation at each stage		Not applicable
		(c) Consider use of a flow diagram		Figure 1

Continued on next page

Descriptive data	14*	(a) Give characteristics of study participants (eg demographic, clinical, social) and information on exposures and potential confounders	9, 10	This was described in the results section and table 1
		(b) Indicate number of participants with missing data for each variable of interest	10 - 14	Number of participants included in the analysis for each variable is described in table 1
		(c) *Cohort study*—Summarise follow-up time (eg, average and total amount)		Follow-up time was defined in the study design and only records of participants with complete follow-up were included in this study (see methods section: "minimum 13 weeks or 90 days if pentavalent antimony, and minimum 26 weeks or 180 days if miltefosine). Number of participants included in each follow-up evaluation is described in Figure 1
Outcome data	15*	*Cohort study*—Report numbers of outcome events or summary measures over time	10	Incidence of failure was estimated at the end of follow-up period. See "Results"
		Case-control study—Report numbers in each exposure category, or summary measures of exposure		
		Cross-sectional study—Report numbers of outcome events or summary measures		
Main results	16	(*a*) Give unadjusted estimates and, if applicable, confounder-adjusted estimates and their precision (eg, 95% confidence interval). Make clear which confounders were adjusted for and why they were included	10 – 14	This is described throughout the results section
		(*b*) Report category boundaries when continuous variables were categorized		This is described in the sections of results and methods
		(*c*) If relevant, consider translating estimates of relative risk into absolute risk for a meaningful time period		Not applicable
Other analyses	17	Report other analyses done—eg analyses of subgroups and interactions, and sensitivity analyses	15, 16	Subgroup and sensitivity analysis were described in the results section, table 3 and S1 Table

Continued on next page

**Discussion**				
Key results	18	Summarise key results with reference to study objectives	16 – 20	This is described throughout the discussion section
Limitations	19	Discuss limitations of the study, taking into account sources of potential bias or imprecision. Discuss both direction and magnitude of any potential bias	20	See discussion section, page 20
Interpretation	20	Give a cautious overall interpretation of results considering objectives, limitations, multiplicity of analyses, results from similar studies, and other relevant evidence	16 – 20	This is described in the discussion section, for all the findings and risk factors identified in the study
Generalisability	21	Discuss the generalisability (external validity) of the study results	20 - 21	Mentioned in the discussion, see: "…which allowed us to generalize our findings to CL patients from central and southwestern Colombia with predominance of *L. V. panamensis*."
**Other information**				
Funding	22	Give the source of funding and the role of the funders for the present study and, if applicable, for the original study on which the present article is based		All sources of funding were declared

*Give information separately for cases and controls in case-control studies and, if applicable, for exposed and unexposed groups in cohort and cross-sectional studies.

Note: An Explanation and Elaboration article discusses each checklist item and gives methodological background and published examples of transparent reporting. The STROBE checklist is best used in conjunction with this article (freely available on the Web sites of PLoS Medicine at http://www.plosmedicine.org/, Annals of Internal Medicine at http://www.annals.org/, and Epidemiology at http://www.epidem.com/). Information on the STROBE Initiative is available at www.strobe-statement.org.
