# Supplementary material for: Risk factors for therapeutic failure to meglumine antimoniate and miltefosine in adults and children with cutaneous leishmaniasis in Colombia: A cohort study
Source: PLoS Negl Trop Dis. 2017 Apr 5;11(4):e0005515. doi: 10.1371/journal.pntd.0005515 (PMC5393627; doi:10.1371/journal.pntd.0005515)
Supplement: S1 Table — (DOCX) [file pntd.0005515.s002.docx]

**S1 Table.** Sensitivity analysis of treatment response at 13 and 26 weeks.

| **Factor** | **Week 13 (n=230)** | | | **Week 26 (n=187)** | | |
| --- | --- | --- | --- | --- | --- | --- |
|  | OR | IC 95% | p | OR | IC 95% | p |
| Treatment with meglumine antimoniate | 3.98 | 1.66 – 9.50 | 0.002 | 3.73 | 1.44 – 9.67 | 0.007 |
| Adherence to the treatment <90% | 3.59 | 1.06 – 12.11 | 0.039 | 19.36 | 3.14 – 119.15 | 0.001 |
| Age ≤ 8 years old | 3.29 | 1.37 – 7.89 | 0.007 | 8.37 | 2.16 – 32.41 | 0.002 |
| Duration of oldest lesion ≤1 month | 2.85 | 1.29 – 6.28 | 0.009 | 3.24 | 1.26 – 8.30 | 0.01 |
| Regional lymphadenopathy | 2.72 | 1.10 – 6.70 | 0.029 | 4.29 | 1.50 – 12.20 | 0.006 |
